# Supplementary material for: In Vitro Discovery of a Therapeutic Lead for HFMD From a Library Screen of Rocaglates/Aglains
Source: J Med Virol. 2025 Feb 8;97(2):e70228. doi: 10.1002/jmv.70228 (PMC11806654; doi:10.1002/jmv.70228)

checkCIF/PLATON report

Structure factors have been supplied for datablock(s) mricca4_autored

THIS REPORT IS FOR GUIDANCE ONLY. IF USED AS PART OF A REVIEW PROCEDURE FOR PUBLICATION, IT SHOULD NOT REPLACE THE EXPERTISE OF AN EXPERIENCED CRYSTALLOGRAPHIC REFEREE.

No syntax errors found. CIF dictionary Interpreting this report

Datablock: mricca4_autored

Bond precision:	C-C = 0.0030 A		Wavelength=1.54184
Cell:	a=11.2346(2)	b=12.3867(3)	c=12.5593(3)
	alpha=105.461(2)	beta=95.6551(18)	gamma=98.1647(19)
Temperature:	100 K		

	Calculated	Reported
Volume	1650.30(7)	1650.30(7)
Space group	P -1	P -1
Hall group	-P 1	-P 1
Moiety formula	C32 H25 F5 O6, C H4 O [+ solvent]	C32 H25 F5 O6, C H4 O, [+ solvent]
Sum formula	C33 H29 F5 O7 [+ solvent]	C33 H29 F5 O7
Mr	632.56	632.56
Dx,g cm-3	1.273	1.273
Z	2	2
Mu (mm-1)	0.921	0.921
F000	656.0	656.0
F000'	658.54	
h,k,lmax	13,14,15	13,14,14
Nref	6037	5878
Tmin,Tmax	0.832,0.832	0.735,1.000
Tmin'	0.832	

Correction method= # Reported T Limits: Tmin=0.735 Tmax=1.000
AbsCorr = MULTI-SCAN

Data completeness= 0.974 Theta(max)= 68.201

R(reflections)= 0.0651(5797) wR2(reflections)= 0.1758(5878)

S = 1.114 Npar= 423

The following ALERTS were generated. Each ALERT has the format
test-name_ALERT_alert-type_alert-level.
Click on the hyperlinks for more details of the test.

Alert level C

```
PLAT029_ALERT_3_C _diffrn_measured_fraction_theta_full value Low .      0.976 Why?
```

Author Response: The somewhat low completion for this triclinic structure could be due

```
PLAT906_ALERT_3_C Large K Value in the Analysis of Variance ......      2.715 Check
```

Author Response: K values were consistent across all resolution ranges.

```
PLAT911_ALERT_3_C Missing FCF Refl Between Thmin & STh/L=    0.600        136 Report
               13  0  0,   0  1  0,   2  1  0,   2  2  0,   2  3  0,  11  6  0,
               10  7  0,  -9 12  0,  -7 13  0,  -5 14  0,  -7-11  1,  -9 -9  1,
               -2 -7  1, -11 -6  1,  13 -2  1,  13 -1  1,  -4  0  1,  -2  0  1,
               13  0  1,   6 11  1,   4 12  1,  -7 13  1,  -6-12  2,  -8-10  2,
               -9 -9  2, -10 -8  2,  13 -2  2,   0 -1  2,  -2  0  2,   0  0  2,
                1  0  2,  -3  1  2,   2  1  2,   0  2  2,   1  2  2,   3  2  2,
               12  2  2,   0  3  2,   1  3  2,   1  4  2,   5 11  2,   3 12  2,
               -6 13  2,  -6-12  3,  -9 -9  3, -10 -8  3,   0 -1  3,   2 -1  3,
                4 -1  3, -13  0  3,  -1  0  3,   1  0  3, -13  2  3, -13  3  3,
              -11  9  3, -10 10  3,  -4 13  3,  -3 13  3,  -4 -2  4,  -1 -2  4,
                2 -2  4,  -1 -1  4,   4 -1  4,  -4  0  4,   0  0  4, -13  2  4,
              -13  3  4, -11  8  4,   1 12  4,   3 -5  5,   3 -4  5,   4 -4  5,
                2 -3  5,  -2 -1  5,  -2  0  5,   2  0  5,   3  2  5, -12  6  5,
              -10  9  5,   2 11  5,  -1 12  5,   4 -1  6, -12  5  6, -11  7  6,
                3 10  6,   0 11  6,  -2 -5  7,  -2 -2  7,  -2 -1  7, -12  3  7,
              -11  6  7,  -1 -5  8,  -1 -6 10,   1 -5 10,   7 -9 11,  -1 -6 11,
```

Author Response: The X8 Proteum has a geometric constraint due to its large detector fa

```
PLAT913_ALERT_3_C Missing # of Very Strong Reflections in FCF ....          4 Note
                0  1  0,   2  1  2,   1  2  2,   1  3  2,
```

Author Response: These reflections may be obscured by the beamstop, or overflows.

```
PLAT918_ALERT_3_C Reflection(s) with I(obs) much Smaller I(calc) .          3 Check
```

Author Response: The observed reflections with I(obs) smaller than I(calc) could be due

Alert level G

```
PLAT042_ALERT_1_G Calc. and Reported MoietyFormula Strings  Differ     Please Check
              Calc: C32 H25 F5 O6, C H4 O
              Rep.: C32 H25 F5 O6, C H4 O,
```

```
PLAT605_ALERT_4_G Largest Solvent Accessible VOID in the Structure        297 A**3
PLAT793_ALERT_4_G Model has Chirality at C9          (Centro SpGr)          S Verify
PLAT793_ALERT_4_G Model has Chirality at C10         (Centro SpGr)          S Verify
PLAT793_ALERT_4_G Model has Chirality at C11         (Centro SpGr)          S Verify
PLAT793_ALERT_4_G Model has Chirality at C25         (Centro SpGr)          R Verify
PLAT793_ALERT_4_G Model has Chirality at C26         (Centro SpGr)          R Verify
PLAT802_ALERT_4_G CIF Input Record(s) with more than 80 Characters          3 Info
PLAT868_ALERT_4_G ALERTS Due to the Use of _smtbx_masks Suppressed          ! Info
PLAT912_ALERT_4_G Missing # of FCF Reflections Above STh/L=  0.600         14 Note
PLAT933_ALERT_2_G Number of HKL-OMIT Records in Embedded .res File          1 Note
                2  3  0,
PLAT961_ALERT_5_G Dataset Contains no Negative Intensities .......     Please Check
PLAT969_ALERT_5_G The 'Henn et al.' R-Factor-gap value ..........      8.853 Note
              Predicted wR2: Based on SigI**2  1.99 or SHELX Weight 15.77
PLAT978_ALERT_2_G Number C-C Bonds with Positive Residual Density.          6 Info
PLAT992_ALERT_5_G Repd & Actual _reflns_number_gt Values Differ by          3 Check
```

---

0 **ALERT level A** = Most likely a serious problem - resolve or explain
0 **ALERT level B** = A potentially serious problem, consider carefully
5 **ALERT level C** = Check. Ensure it is not caused by an omission or oversight
15 **ALERT level G** = General information/check it is not something unexpected

1 ALERT type 1 CIF construction/syntax error, inconsistent or missing data
2 ALERT type 2 Indicator that the structure model may be wrong or deficient
5 ALERT type 3 Indicator that the structure quality may be low
9 ALERT type 4 Improvement, methodology, query or suggestion
3 ALERT type 5 Informative message, check

It is advisable to attempt to resolve as many as possible of the alerts in all categories. Often the minor alerts point to easily fixed oversights, errors and omissions in your CIF or refinement strategy, so attention to these fine details can be worthwhile. In order to resolve some of the more serious problems it may be necessary to carry out additional measurements or structure refinements. However, the purpose of your study may justify the reported deviations and the more serious of these should normally be commented upon in the discussion or experimental section of a paper or in the "special_details" fields of the CIF. checkCIF was carefully designed to identify outliers and unusual parameters, but every test has its limitations and alerts that are not important in a particular case may appear. Conversely, the absence of alerts does not guarantee there are no aspects of the results needing attention. It is up to the individual to critically assess their own results and, if necessary, seek expert advice.

Publication of your CIF in IUCr journals

A basic structural check has been run on your CIF. These basic checks will be run on all CIFs submitted for publication in IUCr journals (*Acta Crystallographica*, *Journal of Applied Crystallography*, *Journal of Synchrotron Radiation*); however, if you intend to submit to *Acta Crystallographica Section C* or *E* or *IUCrData*, you should make sure that full publication checks are run on the final version of your CIF prior to submission.

Publication of your CIF in other journals

Please refer to the *Notes for Authors* of the relevant journal for any special instructions relating to CIF submission.

PLATON version of 15/07/2024; check.def file version of 15/07/2024

Datablock mricca4_autored - ellipsoid plot

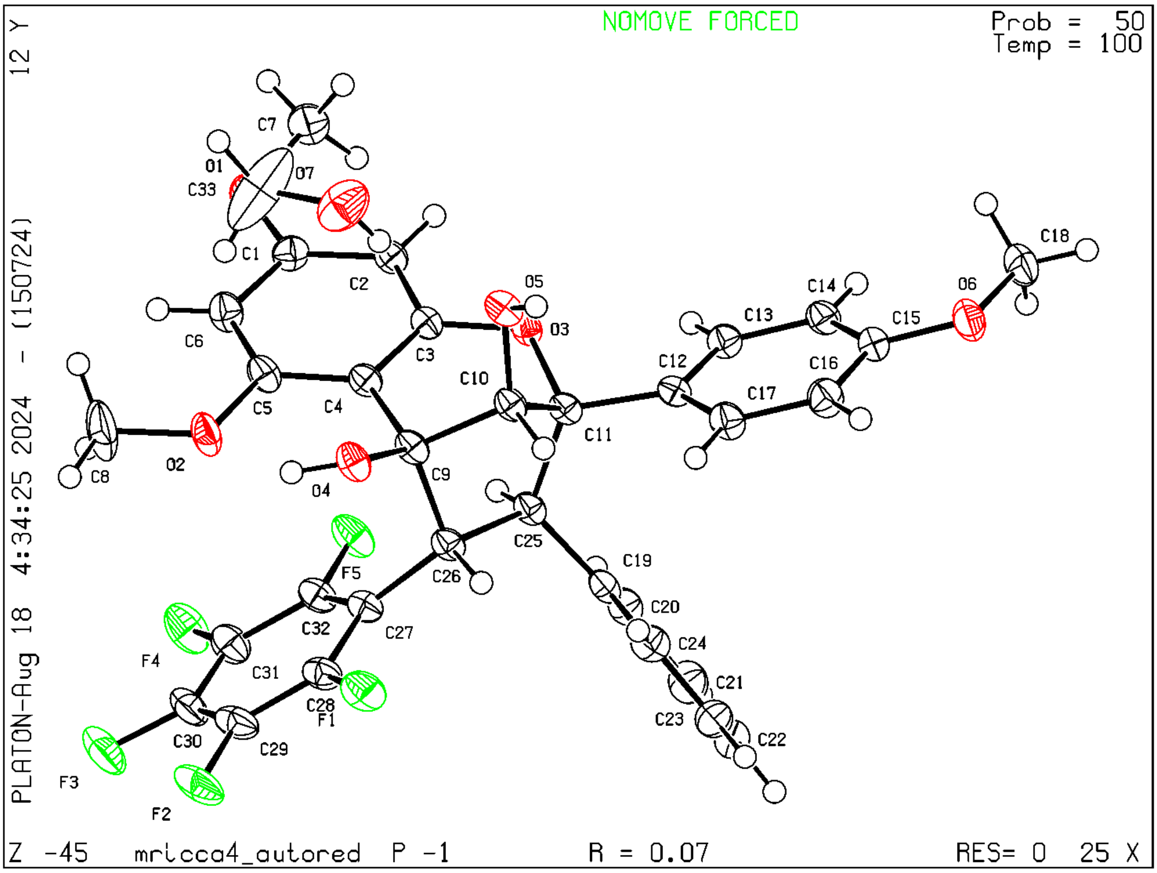

Supplement: Supplementary file 1 — Supporting information. [file JMV-97-e70228-s001.pdf]
